# Structural asymmetry of the insula is linked to the lateralization of gesture and language

**DOI:** 10.1111/ejn.12888

**Published:** 2015-04-10

**Authors:** Szymon P Biduła, Gregory Króliczak

**Affiliations:** 1 Action & Cognition Laboratory, Institute of Psychology, Adam Mickiewicz Universityul. Szamarzewskiego 89, 60-568, Poznan, Poland

**Keywords:** Broca’s area, human, praxis, right-hand, supramarginal gyrus, verbal fluency

## Abstract

The control of gesture is one of the most left-lateralized functions, and the insular cortex is one of the most left-biased structures in the human brain. Therefore, we investigated whether structural asymmetries of the insula are linked to the organization of functional activity during gesture planning. We reconstructed and parcellated the insular cortex of 27 participants. First, we tested 15 strongly left-handed individuals because of a higher incidence of atypical organization of functions such as gesture and language in such a population. The inter-hemispheric structural asymmetries were compared with the lateralization of activity for gesture in the supramarginal gyrus (the hotspot of signal increase regardless of the gesturing hand) and Broca’s area (the hotspot of signal increase for language production). The more pronounced leftward structural asymmetries were accompanied by greater left-hemisphere dominance for both of the studied functions. Conversely, an atypical, bilateral or rightward functional shift of gesture and language was accompanied by an attenuated leftward asymmetry of the insula. These significant relationships were driven primarily by differences in surface area. Subsequently, by adding 12 right-handed individuals to these analyses we demonstrated that the observed significant associations are generalizable to the population. These results provide the first demonstration of the relationships between structural inter-hemispheric differences of the insula and the cerebral specialization for gesture. They also corroborate the link between insular asymmetries and language lateralization. As such, these outcomes are relevant to the common cerebral specialization for gesture and language.

## Introduction

One of the most intriguing characteristics of the human brain is lateralization of its functions. Yet, despite great strides that have been made, our understanding of the causes behind these functional asymmetries remains limited (Toga & Thompson, 2003). It has been suggested, for example, that anatomical differences between the two cerebral hemispheres could reflect or even determine functional lateralization (Geschwind & Galaburda, 1985). Consistent with this proposal, numerous studies have investigated the putative relationships between asymmetries of the planum temporale and/or Broca’s area and language dominance (for an early review, see Shapleske *et al*., 1999). Nevertheless, no clear-cut evidence for associations between the structural hemispheric differences of these regions and language lateralization were shown (Keller *et al*., 2009; cf. Josse *et al*., 2009). Some of the mixed findings could result from examining patients with presumed abnormal structure–function organization (e.g. Foundas *et al*., 1996; Dorsaint-Pierre *et al*., 2006), or the lack of evaluation of both anatomy and function in the same subjects (e.g. Geschwind & Levitsky, 1968; Foundas *et al*., 1998). Most importantly, the absence of success in early studies combining structural and functional asymmetries could have been a consequence of limiting regions of interest to areas commonly associated with language functions.

Current research (Keller *et al*., 2011; Chiarello *et al*., 2013; Greve *et al*., 2013) indicates that the insular cortex should be a prime candidate for closer examination. It is engaged in many tasks (Kurth *et al*., 2010), including planning tool use and more symbolic gestures (Króliczak & Frey, 2009; Vingerhoets *et al*., 2011), and has been also shown to play an important role in speech articulation (Dronkers, 1996; Wise *et al*., 1999). As these functions are strongly lateralized, including the activity of the insula, it raises the possibility that the morphology of its cortex may be paramount to cerebral specializations in general. Consistent with this view, structural asymmetries of the insula have been related to experience with sign language (Allen *et al*., 2008), the lateralization of verbal fluency in right- (Keller *et al*., 2011) and left-handers (Greve *et al*., 2013), as well as to visual-field advantage for word reading (Chiarello *et al*., 2013). Importantly, such relationships were absent for asymmetries of Broca’s area (Keller *et al*., 2011), planum temporale, pars opercularis and pars triangularis (Greve *et al*., 2013).

The search for putative anatomical markers of functional lateralization is of great clinical importance. For example, in the acute phase of stroke, knowledge on asymmetries of crucial structural landmarks could aid in choosing and predicting a proper type, and even the extent, of neurocognitive rehabilitation. Therefore, in this study we aimed to determine whether anatomical asymmetries of the insula could serve as such a marker. In accordance with Keller *et al*. (2011) strong right-hemispheric organization of gesture – a function that is closely related to language – should be associated with changes in the direction of insular asymmetry.

## Materials and methods

### Participants

Twenty-seven anatomical images of the brains of left- and right-handed English native speakers were analysed. The first sample consisted of 15 left-handed individuals (eight females; mean age = 24.8 years, SD = 8.4) who underwent functional scanning during gesture and language tasks (cf. Króliczak *et al*., 2011 for their analyses of gesture and language co-lateralization in the same subjects). All these individuals were in the upper quartile of left-handedness (range from −66 to −100; mean = −90, SD = 9), as indicated by the revised Edinburgh Handedness Inventory (Oldfield, 1971). This sample was of critical importance because from earlier studies we knew that there was large variability in the lateralization of their functions, which is paramount for testing for any correlations between function and structure (Cai *et al*., 2013; Greve *et al*., 2013; see also Króliczak, 2013; Willems *et al*., 2014). Moreover, to ensure that our findings can be generalized to a population at large, we supplemented our analyses for gesture with data from 12 right-handers (six females; mean age = 27 years, SD = 6; cf. Króliczak & Frey, 2009 for their analyses of gesture representations in these subjects). The vast majority of the participants in this group were in the upper quartile of right-handedness, but typically did not show extreme right-hand preference, as evaluated by the Edinburgh Handedness Inventory (Oldfield, 1971): mean Edinburgh laterality index = 85, range: 46–100, SD = 15. None of the participants tested had a history of neurological or psychiatric disorders, and there were no apparent abnormalities in the structures of their brains. The procedures involved in data collection and their processing were approved by the Ethics Committee for Research Involving Human Subjects at the University of Oregon and the Bioethics Committee at Poznan University of Medical Sciences.

### Structural analyses: parcellation of the insular cortex

To investigate structural asymmetries of the insular cortices, we used T1-weighted images acquired with standard magnetization prepared rapid gradient echo pulse sequence (time repetition = 2000 ms; echo time = 4.38 ms; inversion time = 1100 ms; flip angle = 8°; 256 × 256 voxel matrix size; field of view = 256 mm; 176 contiguous axial slices; voxel size = 1 × 1 × 1 mm) on a Siemens (Erlangen, Germany) 3-T Allegra magnetic resonance imaging scanner.

Cortical reconstruction and parcellation of the insula was performed with the recon-all procedure implemented in freesurfer 4.9. All details of this routine are described in the following publications (Dale *et al*., 1999; Fischl *et al*., 1999a, 2002, 2004; Fischl & Dale, 2000; Ségonne *et al*., 2004; Jovicich *et al*., 2006). This procedure involves averaging multiple structural images to increase signal-to-noise ratio. Next, to account for the presence of intensity fluctuations within the tissues, bias field correction with the N3 algorithm was carried out (Sled *et al*., 1998; Zheng *et al*., 2009). Subsequently, the Talairach affine transform was computed, and skull stripping was performed using the Hybrid Watershed Algorithm (Ségonne *et al*., 2004). The resulting images were the basis for tissue segmentation (Dale *et al*., 1999). To perform cortical reconstruction the midbrain was removed from the cerebrum and the two hemispheres were separated using two cut planes. Then, each hemisphere white/grey matter boundary was tessalated and smoothed. After automated topology correction (Fischl *et al*., 2001; Segonne *et al*., 2007) the original surface was nudged to follow the white–grey matter and, separately, grey matter – cerebrospinal fluid (CSF) intensity gradients, thus resulting in the white and pial surfaces, respectively. Subsequently, these corrected surfaces were registered to a surface-based coordinate system (Fischl *et al*., 1999b) created from 40 control subjects (Buckner *et al*., 2004) for anatomical parcellation (Fischl *et al*., 2004). This cortical segmentation could be performed by virtue of different criteria, e.g. using exclusively gyral or sulcal features, as in Desikan *et al*. (2006), or a mixed sulco-gyral parcellation as in Destrieux *et al*. (2010). The latter approach was adopted in this study to segment the insular cortex.

**Figure 1:**
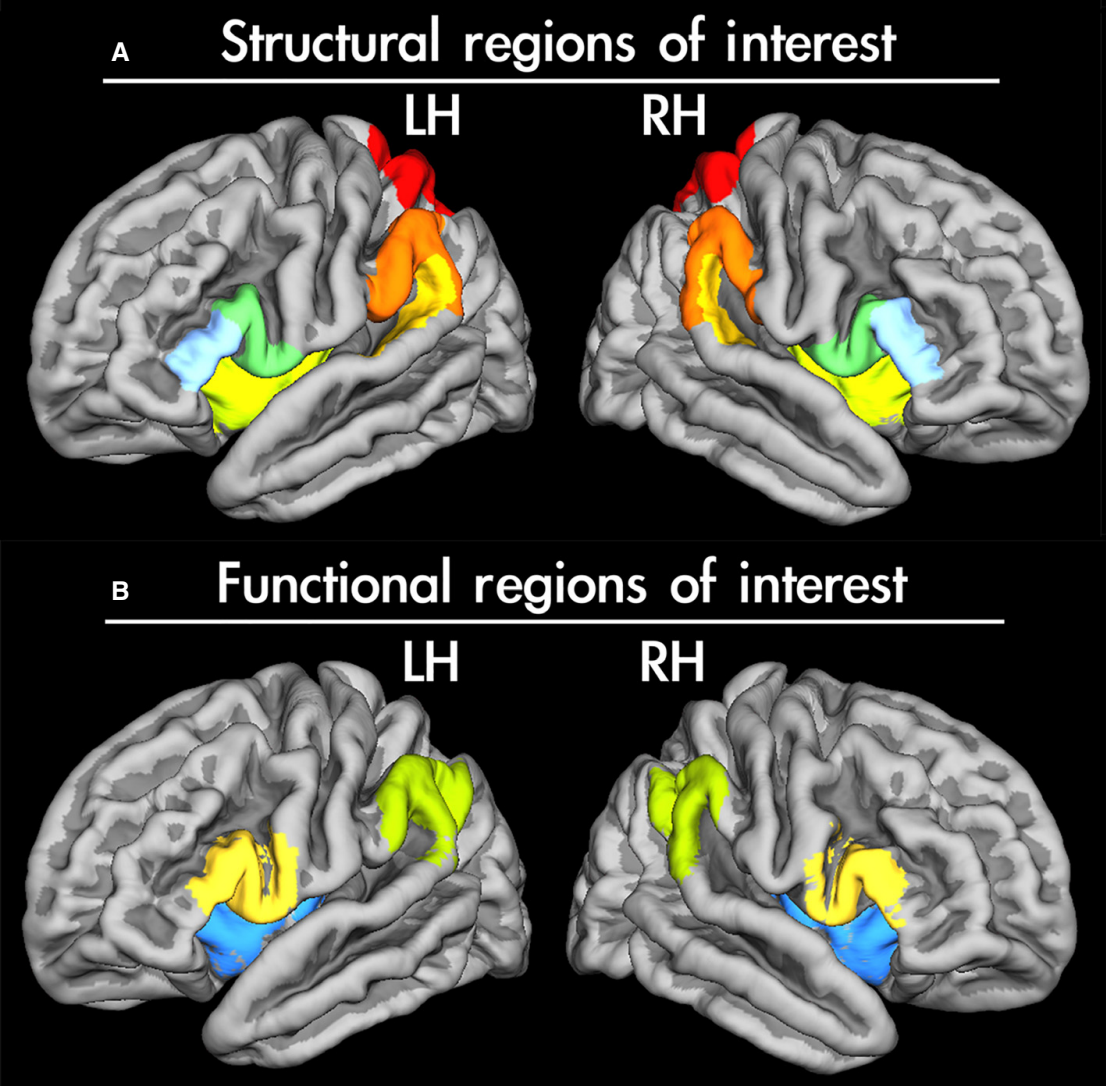
Regions of interest (ROIs) shown on the average cortical surface of all left-handed participants involved in this study. (A) ROIs used for structural analyses. The maps were derived from the Destrieux *et al*. (2010) atlas implemented in FreeSurfer. Colour codes: light blue denotes pars triangularis; pale green, pars opercularis; light green and pale yellow, insula; dark yellow, planum temporale; orange, supramarginal gyrus; red, superior parietal lobe. (B) ROIs used for analyses of the lateralization of functional activity. The binarized and bilateral maps of Broca’s area defined as BA44/45 (depicted in yellow), supramarginal gyrus (green) and insular cortex (blue). These stereotaxic maps were thresholded (to include only voxels with at least a 50% chance of belonging to a respective ROI) and binarized. These probabilistic masks were obtained from the Jülich histological atlas (Broca’s area, supramarginal gyrus; Amunts *et al*., 1999; Caspers *et al*., 2006; Eickhoff *et al*., 2007) or from the Harvard–Oxford atlas (insular cortex; Desikan *et al*., 2006) implemented in FSL.

In the Destrieux *et al*. (2010) atlas, the insula is in fact divided into five subunits. To first obtain one representation of the whole insular cortex – our structural region of interest (ROI) in each hemisphere – we added all five parcellations: the long insular gyrus and insular central sulcus, the short insular gyrus, the anterior segment of the circular sulcus of the insula, the inferior segment of the insular circular sulcus, and finally the superior segment of the insular circular sulcus. The combined subdivisions then served as the structural ROI of the insular cortex, which is depicted in Fig.1A.

Based on this ROI we extracted information on the cortical volume of this structure, the overall surface area (i.e. the area of a vertex on the gray matter surface, calculated as the average of the area of the triangles touching that vertex), and the average cortical thickness (i.e. the closest distance from the grey/white boundary to the grey/CSF boundary at each vertex on the tessellated surface). These three variables were then used to investigate the relationships between anatomical asymmetries of the insular cortex and the functional lateralization of gestures and language. Structural asymmetries, expressed as laterality indices (LIs), were calculated according to the formula: LI = [(L − R)/(L + R)] × 100 where L (left) and R (right) reflect the cortical volume, insular surface area or cortical thickness in the left or right hemisphere.

Structural inter-hemispheric differences were also assessed in the following five regions: the pars opercularis, pars triangularis, planum temporale, supramarginal gyrus and superior parietal cortex. These structural ROIs were chosen based on – (i) previous reports of their asymmetry (the planum temporale), (ii) their involvement in gesture planning (the supramarginal gyrus, superior parietal lobule) and (iii) an engagement in language tasks (pars opercularis, pars triangularis). Two-tailed, Bonferroni-corrected *t*-tests were used to determine significance of the inter-hemispheric differences of the surface area and cortical thickness.

Moreover, we performed a vertex-wise search for any local asymmetries that would not be observable at the level of the whole ROIs. This procedure entails resampling of the studied cortical surfaces to a symmetrical atlas, which enables a comparison of the left and right hemisphere. We chose the fsaverage_sym template distributed with the FreeSurfer package (see Greve *et al*., 2013 for details of the atlas creation). After registering each surface to the common space, smoothing using a 10-mm kernel was applied. Then left and right hemispheres where compared for surface and thickness asymmetries. The results were thresholded (*P* < 0.05), and corrected for multiple comparisons with a false discovery rate (FDR) procedure (Genovese *et al*., 2002).

### Functional analyses: gestures and language laterality measurements

To investigate functional asymmetries of gesture and language representations, we used T2*- weighted gradient echo sequence images acquired with the following parameters: time repetition = 2000 ms; echo time = 30 ms; flip angle = 80°; 64 × 64 matrix; field of view = 200 mm; 33 contiguous axial slices, 3.0-mm isotropic voxels. FSL 4.1.4 was utilized to perform all the necessary preprocessing steps, including reduction of non-neural variance, and to run whole-brain analyses to obtain statistical parameter maps [*Z*], as described in detail by Króliczak & Frey (2009). Gesture planning activity in each condition was modelled as the 3.5-s period beginning with the onset of the cue (hammering, wavering, etc.) and lasting through the end of the shortest (2-s) delay interval where the 1.5-s instructional cue was followed by a variable delay (2, 4, or 6-s) interval during which gestures were planned (see Króliczak & Frey, 2009 for further details). In the language laterality test, a 30-s instructional cue in the form of a single letter, such as M, G, E, T, H and L, indicated that participants should silently generate as many words as possible starting with the presented letter. Such blocks were then followed by 30-s rest periods with which they were contrasted in statistical analyses (for further details see Króliczak *et al*., 2011).

Subsequent laterality assessments were confined to ROIs. The ROIs were defined by means of probabilistic cytoarchitectonic maps of Brodmann areas (BAs) implemented in the Jülich histological atlas (Eickhoff *et al*., 2007), a component of the FSL package. Prior to all analyses and delineation of ROIs, these maps were thresholded at 50% probability value. The first ROI, BA 40, for the assessment of lateralization of gesture planning, was delimited by PF and PFm divisions of the inferior parietal lobule (IPL) (Caspers *et al*., 2006), which approximates the supramarginal gyrus (Króliczak *et al*., 2011). The second ROI, BA 44/45, which approximates Broca’s area, was used for evaluating language lateralization (Amunts *et al*., 1999; Króliczak *et al*., 2011). The third ROI, insular cortex, was derived from the Harvard–Oxford atlas also implemented in the FSL package. The three ROIs for the functional data are shown in Fig.1B.

Task-related asymmetries in the functional maps from both tests were measured in a manner proposed by Jansen *et al*. (2006). In short, first for each subject and each ROI, activity maps were thresholded at 95% of the maximum *Z* value and the average activity of the voxels that survived was calculated. The obtained value was then used as a reference for thresholding the original activity map at 50% of this value. Subsequently, all the voxels that passed the thresholding were counted. Functional LIs were then calculated again according to the formula: LI = [(L − R)/(L + R)] × 100 where L (left) and R (right) stands for the number of voxels that exceeded a particular threshold cut-off in the respective ROI. Mean LI values across all thresholds were used as markers of functional lateralization of gestures, and language. These indices can range from +100 to −100, with 0 indicating an equal number of activated voxels in the left and right ROIs. The values of +100 to +33.3 indicate a strong to weak left-hemispheric dominance, and −33.3 to −100 reflect a weak to strong right-hemispheric dominance (Króliczak *et al*., 2011).

### Statistical analyses

To test whether there are links between structural asymmetries of the insula and the lateralization of gestures (and as well as for language in our control task in left-handers), we analysed correlations between the LI for structure and functions using Pearson’s correlation coefficients (*r*). All the described statistical analyses were conducted with spss version 22.0. Unless stated otherwise, the reported correlations were considered significant at the 0.05 level, two-tailed.

## Results

### Structural analyses in left-handed participants

**Figure 2:**
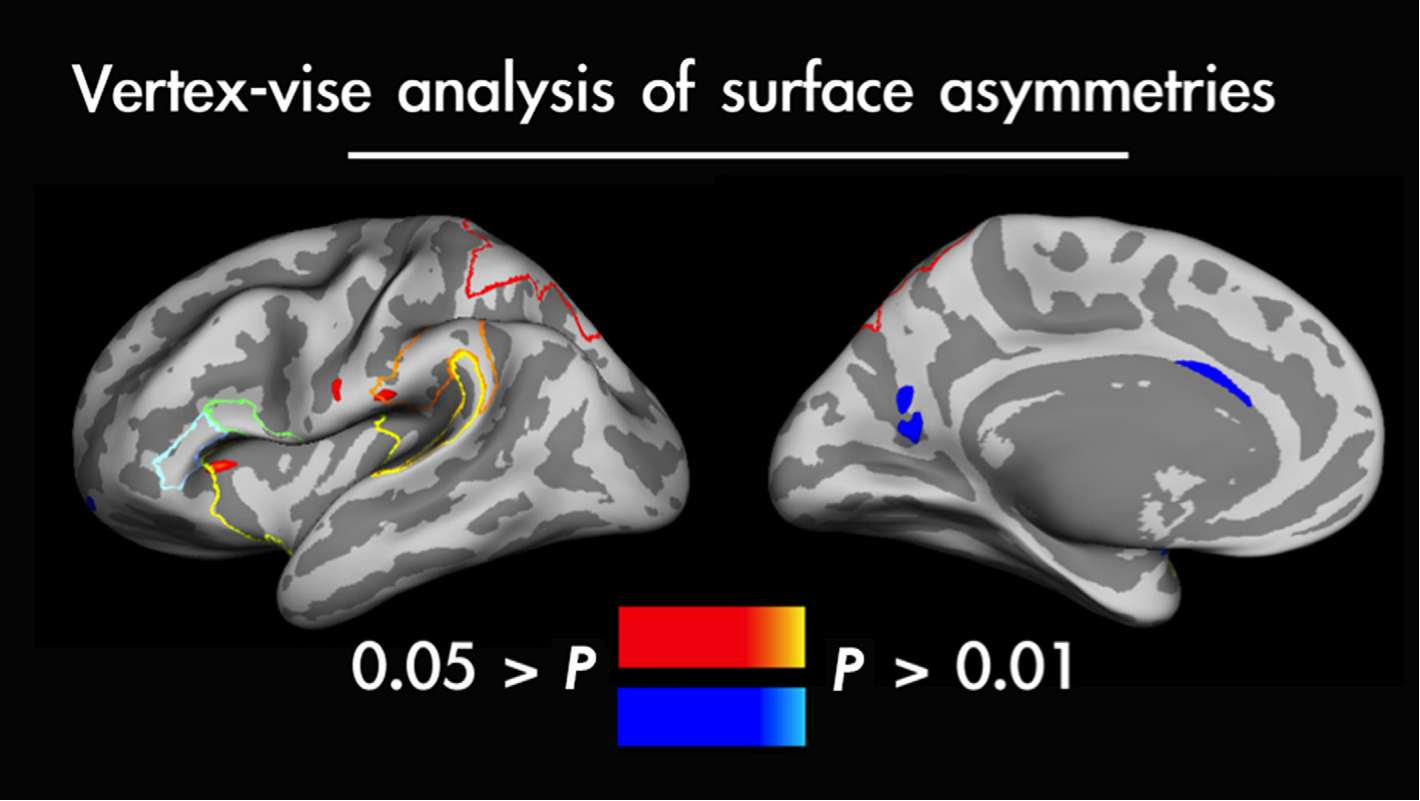
Vertex-wise analysis of surface asymmetries measured across the whole brain shown on an inflated average brain of all study participants. Significant leftward surface asymmetries (depicted in warm colours) were located in the insular cortex – a structure of primary interest for the present study – supramarginal gyrus and post-central gyrus. Significant rightward surface asymmetries were present inferiorly in the parieto-occipital sulcus, and the anterior part of the cingulate gyrus.

The only significant differences between surface areas were observed in the left and right insula (*t*_14_ = 8.04; *P* = 0.000001), and the left and right planum temporale (*t*_14_* = *4.72; *P* = 0.00033). We found no evidence of any asymmetries for cortical thickness in any ROIs considered. To corroborate that the effects observed within the selected structural ROIs are not accidental we ran a vertex-wise analysis (i.e. confined to cortical surface only) across the whole brain (*P* < 0.05, FDR-corrected). Here, consistently with the ROI analyses (performed separately in each participant), at a group level analysis we did find clusters of significant leftward surface asymmetries located within the insular cortex, but not in the planum temporale. Interestingly, this whole brain analysis revealed a leftward asymmetry in the supramarginal gyrus. All in all, only the insular surface exhibited significant leftward asymmetry in both analyses (mean LI for structural ROI = 5.91, SD = 3.02; a significant difference in cluster size = 0.05 > *P *>* *0.01 for vertex-wise analysis in favour of the left side). The results of voxel-wise comparisons are depicted in Fig.2.

### Functional analyses

#### Gesture planning

**Figure 3:**
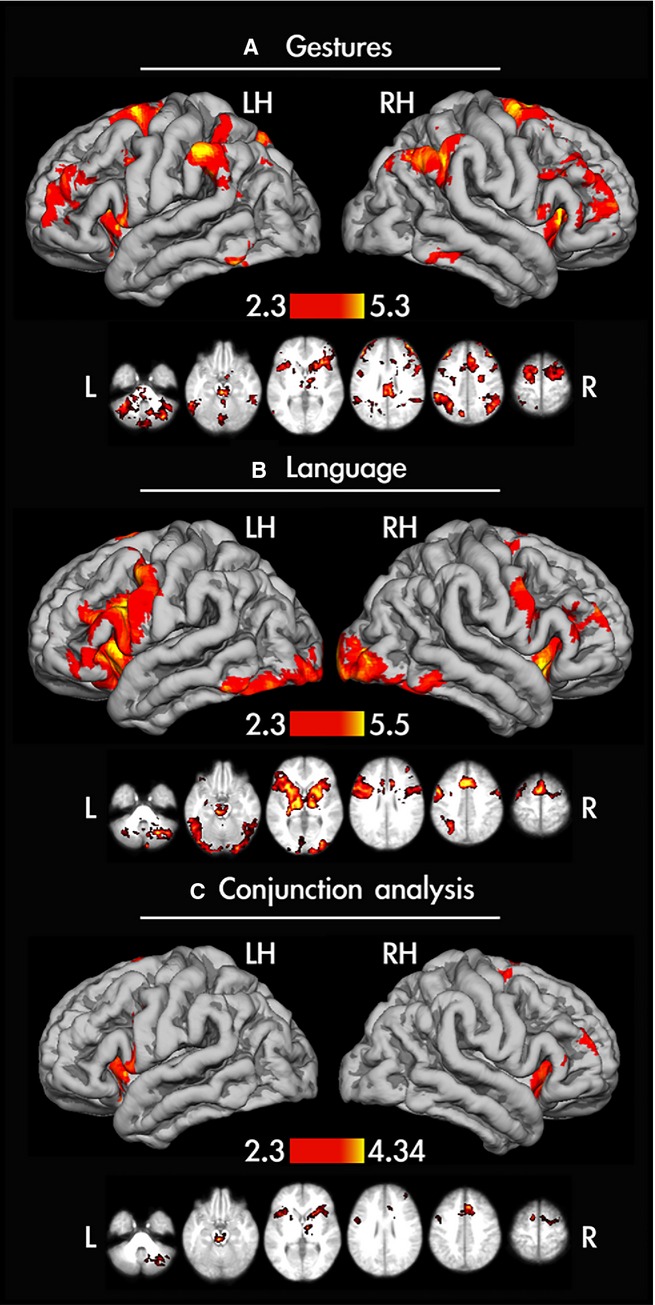
Gesture- and language-related activity shown on the mean cortical surface of all left-handed participants’ brains. (A) Functional activity for gesture planning averaged across hands and gestures. In the majority of participants this activity was largely symmetrical, with the exception of the anterior supramarginal gyrus (SMG), anterior intraparietal sulcus (aIPS) and the superior parts of the superior parietal gyrus (SPG), where it was exclusively on the left. The bilateral involvement was observed in the insular cortex, the ventral premotor and dorsal premotor cortices, the supplementary motor complex (SMA complex), the middle frontal gyrus, the inferior temporal gyrus and the cerebellum. The angular gyrus activity was modulated on the right. (B) Functional activity in a language task (verbal fluency). Broca’s area, and insular and orbitofrontal cortices were engaged primarily on the left, whereas the fundus of aIPS was engaged exclusively on the left. The SMA complex, the anterior cingulate cortex and the majority of subcortical structures were modulated bilaterally. Given that the contrast reported was against the resting baseline, there is also a clear bilateral involvement of striate and extrastriate visual areas. (C) A conjunction test showing areas involved independently of the task (across gesture and language). Except for the middle frontal involvement on the right, the insular cortex and the SMA complex were engaged bilaterally. The small cluster of ventral premotor activity was exclusively left lateralized and the dorsal premotor cluster was exclusively right lateralized.

In the first level group analysis we identified regions active for planning either type of gestures for each hand with linguistic processing accounted for. To this end, first we averaged neural responses for planning transitive and intransitive gestures for both hands across each participant (using a fixed effects analysis), and then collapsed across all studied subjects (using a mixed effects analysis). This analysis revealed widespread bilateral activity of frontal, temporal and parietal cortices, with left hemisphere prevalence in nearly half of the participants. As can be seen in the group data shown in Fig.3A, there was the expected significant involvement of the supramarginal gyrus (SMG), the intraparietal sulcus (IPS) and superior parietal gyrus (SPG), with their anterior divisions activated exclusively in the left hemisphere. The dorsal and ventral premotor cortices (PMd, PMv), anterior parts of the insular cortex, rostral middle frontal gyri (rMFG), supplementary and pre-supplementary motor cortices (SMA complex), the left caudal inferior temporal gyrus (cITG) and anterior divisions of the fusiform gyrus (FusiG) were involved bilaterally. The signal modulations within the angular gyrus (ANG) were found only on the right.

#### Verbal fluency

Silent generation of words beginning with a particular letter compared with resting baseline resulted in increased activation in the bilateral insula, IFG, rMFG, PMv and SMA. Moreover, significant activity was also found in the ventral parts of the pre-central gyrus (more pronounced in the left hemisphere), in the left anterior IPS (aIPS), in the left and right caudal ITG, and more posteriorly in the lateral occipital cortex, and on the medial surface in the vicinity of the calcarine sulcus. The outcomes of this analysis are shown in Fig.3B.

#### Areas common to gesture planning and language

The results from the comparison of the minimum statistic to the single image threshold (i.e. a more conservative approach, as suggested by Nichols *et al*., 2005) revealed that both gesture planning and word generation significantly engaged the bilateral insula, PMv, SMA complex and right rMFG. These outcomes are depicted in Fig.3C.

#### Gesture planning lateralization

**Figure 4:**
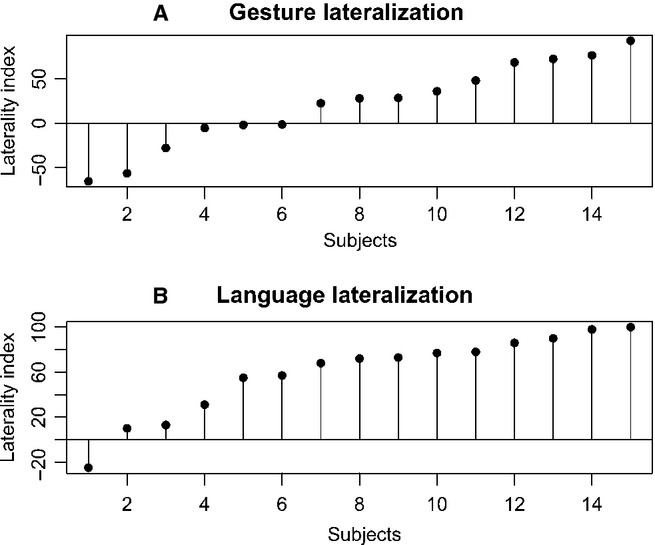
Laterality indices (LIs) for each left-handed participant. (A) LIs for gesture measured in the supramarginal gyrus (BA40). For display purposes individual LIs were sorted in ascending order. (B) LIs for language assessed in Broca’s area (BA44/45), presented in ascending order.

When SMG was treated as one ROI, without differentiating between its anterior and posterior divisions, nearly half of the participants (47%) showed bilateral representation of gesture planning in SMG. Nevertheless, there was also a substantial group of subjects with the left-hemisphere organization of this function (40%) in the same ROI. The remaining 13% of individuals exhibited right hemisphere organization of gesture. These results are depicted in Fig.4A.

When the whole hemisphere was treated as an ROI, all participants demonstrated equal distribution of activity during gesture planning. Yet, there was still a significant correlation between LIs as measured in the SMG and at the whole hemisphere level (*r*_13_ = 0.86, *P* = 0.000047). In the insular cortex, 67% of the participants demonstrated bilateral organization, 20% of the subjects exhibited right-hemisphere dominance, and only 13% showed the left-hemisphere lateralization for gesture planning. Still, there was a significant correlation between the LIs as assessed in the insular cortex and in the SMG (*r*_13_ = 0.47, *P* = 0.04, one-tailed). Finally, a trend towards significance was observed between the LIs measured in the insular ROI and at the level of the whole hemisphere (*r*_13_ = 0.42, *P* = 0.119).

#### Language lateralization

As expected and revealed by LIs, most of the studied individuals (73%) demonstrated left-hemispheric advantage, whereas the remaining subjects exhibited bilateral organization of language within Broca’s area (BA 44/45) during silent word generation. The results are shown in Fig.4B. In contrast, at the level of the whole hemisphere, the majority of participants (80%) showed bilateral representation of language, and the remaining individuals demonstrated left-hemispheric advantage. Similarly, when language lateralization was measured in the insular cortex, most of the subjects (73%) exhibited bilateral representation of this function. Among the remaining cases, there were two individuals with left-hemispheric representation of language, and two individuals with right-hemispheric dominance of this function.

Interestingly, all LIs (irrespective of the ROI from which they were derived) correlated with each other. Specifically, LIs as measured at the level of the whole hemisphere were linked to the LIs assessed in Broca’s area (*r*_13_ = 0.87, *P* = 0.000027) and insular cortex (*r*_13_ = 0.49, *P* = 0.033, one-tailed). Moreover, the laterality scores obtained in Broca’s area were significantly correlated with those from the insular cortex ROI (*r*_13_ = 0.6, *P* = 0.018).

#### Structural asymmetry of the insula and functional LIs

**Figure 5:**
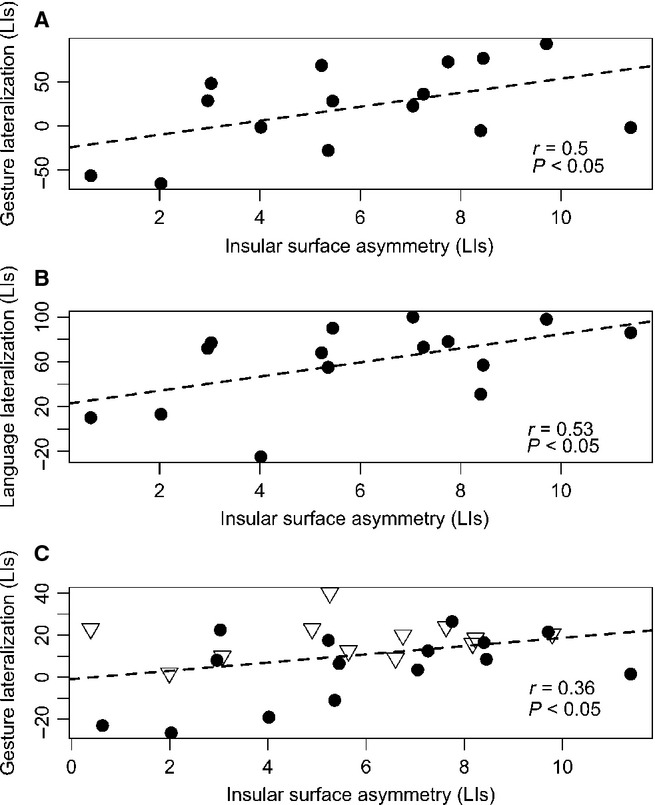
Correlations between the insular surface asymmetries and the lateralization of functional activity for gesture and language. (A) Gesture lateralization and insular surface asymmetries. There was a significant correlation between their respective LIs. (B) Language lateralization and insular surface asymmetries. The lateralization of activity in Broca’s area was significantly correlated with the insular surface asymmetry, both again expressed in LIs. (C) Gesture lateralization, measured at the whole hemisphere level, and insular surface asymmetries. Likewise, there was a significant correlation between gesture LIs and insular surface LIs. Black dots represent left-handers, whereas triangles represent right-handers.

The most critical comparisons for this project revealed that there were significant relationships between the anatomical asymmetries of the insular cortex and the lateralization of functional activity for both gesture and language. Namely, consistent with our hypothesis, we observed significant relationships between insular volume asymmetry and gesture LIs measured in SMG (*r*_13_ = 0.6, *P* = 0.02), and between insular volume asymmetry and language LIs measured in Broca’s area (*r*_13_ = 0.49, *P* = 0.03). Having the possibility to investigate these relationships further, we examined whether these effects were driven by hemispheric differences in the extent of insular surface rather than the thickness of its cortex. As volume is more closely related to surface area (Winkler *et al*., 2010; in our data the LIs for volume and surface were also significantly correlated; *r*_13_ = 0.89, *P* = 0.000011), the outcomes may indeed be driven primarily by surface asymmetries. This was in fact the case both for gesture measured in SMG (*r*_13_ = 0.50, *P* = 0.03, one-tailed) and language laterality assessed in Broca’s area (*r*_13_ = 0.53, *P* = 0.04). These significant relationships are depicted in Fig.5A and B, respectively.

Interestingly, we have found even stronger effects when these relationships were assessed in the whole hemisphere functional ROI: there were significant correlations between individual asymmetries of the insular surface area and the lateralization of gestures (*r*_13_ = 0.54, *P* = 0.04), as well as significant correlations between the insular surface asymmetry and the lateralization of language (*r*_13_ = 0.66, *P* = 0.008) evaluated across the whole hemisphere.

Thickness asymmetries for the insular cortex, by contrast, correlated neither with gesture LIs measured in SMG (*r*_13_ = 0.11, *P* = 0.66) nor with language LIs assessed in Broca’s area (*r*_13_ = 0.09, *P* = 0.75). Neither did we find any significant correlations between asymmetries of the insular thickness and gesture (*r*_13_ = 0.04, *P* = 0.881), or language (*r*_13_ = 0.07, *P* = 0.81) as assessed in the whole hemisphere ROI.

Finally, insular surface asymmetries showed significant correlations with the functional asymmetries of activity for gesture and language in the insula itself (*r*_13_ = 0.49, *P* = 0.033; *r*_13_ = 0.47, *P* = 0.038; respectively, one-tailed). The surface asymmetries of the planum temporale, by contrast, did not correlate either with gesture measured in the SMG (*r*_13_ = 0.03, *P* = 0.92) and at the hemispheric level (*r*_13_ = −0.10, *P* = 0.72) or with language assessed in Broca’s area (*r*_13_ = −0.2, *P* = 0.47) and at the whole hemisphere level (*r*_13_ = −0.24, *P* = 0.39). Thus, these results corroborate specificity of the above findings.

### Structural asymmetry of the insula and functional LIs for gestures in all 27 individuals

To demonstrate that the results obtained for gesture and insular asymmetry in left-handers can be generalized to the population, the analyses of the brains of the 15 left-handers were extended to additional 12 right-handed individuals. This is justified because the majority of our left-handed participants, similarly to right-handers (especially for the control of the left-hand, routinely tested in patients in clinical settings), demonstrated the typical left-sided activity in anterior divisions of IPL (consistent with Vingerhoets *et al*., 2012; see also Króliczak & Frey, 2009). As far as insular surface is considered, the correlations with functional activity in the SMG ROI (*r*_25_ = 0.29, *P* = 0.07, one-tailed) as well as in the functional insular ROI (*r*_25_ = 0.32, *P* = 0.06, one-tailed) showed only clear trends towards significance. Nevertheless, we did observe significant correlations between the surface asymmetries of the insular cortex and the lateralization of functional activity for gesture measured at the level of the whole hemisphere (*r*_25_ = 0.36, *P* = 0.032, one-tailed). This significant relationship is depicted in Fig.5C.

Finally, it should be emphasized that much stronger and significant effects were observed when the whole insular volume was considered. Specifically, insular volume asymmetries correlated significantly with the laterality of activity for gesture assessed both in SMG (*r*_25_ = 0.45, *P* = 0.018) and at the whole hemisphere level (*r*_25_ = 0.40, *P* = 0.036).

## Discussion

This study demonstrates, to our knowledge, for the first time that asymmetries of the insular cortex can be linked to the lateralization of functional activity accompanying gesture. Moreover, consistent with earlier studies (Keller *et al*., 2011; Chiarello *et al*., 2013; Greve *et al*., 2013), we also show that such asymmetries are linked to the lateralization of language. Namely, the more pronounced leftward structural asymmetries were accompanied by greater left hemispheric dominance for both the studied functions. Conversely, atypical, i.e. mainly bilateral, organization of both gesture and language was related to an attenuated leftward asymmetry of the insula. As such, these results are consistent with a study by Van Essen *et al*. (2012) who demonstrated that one of the left-biased hotspots of surface asymmetries in the human cerebral cortex is located in the insula. Yet, by simultaneous examination of both structural and functional differences between the two hemispheres, we considerably extend their conclusions by linking such insular volume and surface asymmetries to the functional organization of gesture, and language. All in all, these outcomes suggest that the structure of the insula may play a pivotal role in the laterality of brain functions.

### Insular cortex asymmetry

Our findings of clear insular cortex asymmetries across left- and right-handed individuals are in agreement with previous studies indicating that at a population level the left insula is greater than its right counterpart (Van Essen *et al*., 2012; Chiarello *et al*., 2013; Greve *et al*., 2013). The uncovered differences in morphology between the left and right insulae is also consistent with several earlier studies reporting insular asymmetries for grey/white matter ratio (Allen *et al*., 2003), and for ‘amounts’ (the number) of grey matter (voxels) in its superior and inferior parts (Watkins *et al*., 2001). Although it is not clear to what extent such structural differences are inherent or experience-driven, there is evidence that some inter-hemispheric insular asymmetries can be observed as early as at birth, with greater insular surface in the left hemisphere (Li *et al*., 2014). Moreover, a recent study utilizing diffusion tensor imaging found that in neonates white matter pathways in the vicinity of the insular lobe also exhibit leftward asymmetry (Ratnarajah *et al*., 2013). This morphological difference between the left and right insulae is also present in the adult brain (Jakab *et al*., 2012). If this initial structural organization determines future lateralization of functions then the magnitude of this asymmetry could be a major factor contributing to the degree of laterality of gestures to the left hemisphere. Yet, experience with manual gesturing also seems to play a modulatory role in shaping such asymmetries, especially when ‘gestures’ are the prime modality for communication, as in sign language. Namely, larger left–right differences in insular volume have been shown to accompany fluent usage of sign language in right-handed deaf individuals (Allen *et al*., 2008) who presumably have their language skills left lateralized. Conversely, atypical cases of the organization of functions could stem from the attenuated, non-existent or even reversed bias in this structure.

### Insular cortex asymmetry and the lateralization of gesture

Our main results clearly indicate that insular cortex asymmetry can be linked to the functional lateralization of gesture. Specifically, we found that the structural asymmetries of the insula – as measured by the extent of its surface, but also by its volume – are strongly related to functional asymmetries in the supramarginal gyrus, a structure typically associated with the control of gesture, as well as to functional asymmetries measured at the whole hemisphere level.

The precise role of the insula in the higher-order motor functions that we studied is currently unknown. In fact, it is not an exaggeration to say that despite a dependence of the manual (Kertesz & Ferro, 1984) and oral gestures (Tognola & Vignolo, 1980) on the insular cortex, its contribution to the lateralization of skilled actions (praxis) has been largely ignored. Not surprisingly, then, the morphology of the insula has never been considered in the context of gesture organization. Perhaps its structural and functional role has been overlooked because any deficiencies in the control of gesture have often been accompanied with lesions to the inferior frontal gyrus. Nevertheless, damage to the left insular cortex can substantially disrupt pantomimed tool use to verbal commands (Goldenberg *et al*., 2007; Buxbaum *et al*., 2014), gesture identification (Pazzaglia *et al*., 2008b) and even the recognition of the buccofacial-related action sounds (Pazzaglia *et al*., 2008a). If the relevant function is controlled by the right hemisphere, it is the damage to the right insula that also causes higher-order motor deficits such as oral and ideomotor apraxia, even in a right-hander (Berthier *et al*., 1987). These cases clearly show that in neuropsychology of action the insular cortex has not received the attention it deserves. For example, in two recent neuroimaging studies showing engagement of the insula in gesture-related functions (Króliczak *et al*., 2011; Vingerhoets *et al*., 2013) its role in higher-order motor control was not considered at all (with the majority of functional studies pointing primarily to the left parietal cortex; for a review see Niessen *et al*., 2014; and also Orban & Caruana, 2014). Our current report shows convincingly there are clear indices that the role of the insula is also worth examining in the context of gesture.

The relationships between insular asymmetries and gesture lateralization are not specific to left-handers but are handedness-independent (see also Chiarello *et al*., 2013). Consistent with this assumption, our results were nearly as clear-cut when the analyses were extended to right-handers, with insular volumes becoming much better indices of such relationships. It is not surprising, however, that the observed relationships changed slightly because, similarly to the general population of right-handers, our sample was characterized by smaller variability in functional lateralization (e.g. Cai *et al*., 2013; Willems *et al*., 2014). By the same token there should be, and indeed in our sample there was, less variability in their structural asymmetries. In other words, greater functional and structural variability observed in left-handers is an asset because it is more likely to show an effect of interest, which can later be generalized to the population at large.

One may speculate that if our right-handers were characterized by an extreme right hand preference (see Chiarello *et al*., 2013), the results would more closely resemble that of consistent left-handers whose data were initially considered here in isolation. By collapsing across right- and left-handers we were able to demonstrate that the relationships observed for gesture are universal (i.e. are not handedness-dependent), and more importantly that there are some parallels with the laterality of language. Namely, our outcomes are similar to those of Chiarello *et al*. (2013) who found correlations between insular asymmetries and the processing underlying lateralized word recognition in right- and left-handers. Indeed, similarly to Keller *et al*. (2011), Chiarello *et al*. (2013) and Greve *et al*. (2013) we did observe associations between insular structural asymmetries and the laterality of language processing, as well.

### Insular cortex asymmetry and the lateralization of language

Before the seminal study by Keller *et al*. (2011), who demonstrated that the language-dominant hemisphere could be predicted by insular asymmetries, virtually no anatomical studies had looked at this structure in this context. Here, we also corroborate that the left-sided asymmetry of the insular surface, as well as the asymmetry of its volume, correlates well with the lateralization of language (i.e. verbal fluency). None of the brains studied in our sample exhibited rightward surface asymmetry of the insular cortex. Thus, unlike Keller *et al*. (2011) we would not have been able to predict language dominance by simply looking at the direction of the insular asymmetry (i.e. that rightward asymmetry would predict right-hemispheric language organization or vice versa). In this respect, our results are similar to those of Greve *et al*. (2013), who also did not observe any rightward asymmetry of the insula in the group with atypical language organization. The possible reason for the apparent discrepancies with Keller *et al*. is that, similar to the work of Greve *et al*. (2013), the majority of participants studied here were strongly left-handed, and had language representations lateralized primarily to their left hemispheres, whereas Keller *et al*. (2011) also included a rare sample of right-handed individuals with right-hemisphere language organization. In sum, we did not find any evidence that subjects with right (or even bilateral) hemispheric language organization also have rightward insular surface asymmetry. Thus, consistent with Greve *et al*. (2013), who used similar methodology and investigated an analogous population, our results showed that atypical laterality of language (and gesture) accompanies greatly attenuated insular asymmetry.

The relationships we observed between structure and function were significant only for the insula. They did not hold for the asymmetries of the supramarginal gyri, superior parietal gyri and subdivisions of the inferior frontal gyri (i.e. pars opercularis and pars triangularis) where the inter-hemispheric differences were negligibly small, and there were no relationships with the laterality of functions whatsoever. Interestingly, although the planum temporale exhibited some significant surface area asymmetry, it was related neither to the lateralization of gesture nor to language. The latter result is consistent with two earlier reports (Keller *et al*., 2011; Greve *et al*., 2013), which did not find any links between the asymmetry of the planum temporale and the functional laterality of language, either.

Lesions to the insular cortex, particularly to its left superior precentral gyrus (Dronkers, 1996) often result in apraxia of speech (Dronkers, 1996; cf. Hillis *et al*., 2004). This impairment is characterized mainly by the inability to coordinate sequential movements of the speech apparatus (Ogar *et al*., 2005). Neuroimaging studies support these results by suggesting that articulation plans are formulated in the left anterior insula (Wise *et al*., 1999). Insular contribution to speech has been also conceptualized as a ‘code translator’ between higher-order aspects of language and the coordination of the actual vocalization (Eickhoff *et al*., 2009). As indicated by Keller *et al*. (2011), and our current results, these multiple roles may also be reflected in insular morphology.

### The role of insular cortex in common cerebral specialization for gestures and language

The putative common cerebral specialization for gestures and language (Kimura, 1982; Króliczak *et al*., 2011; Vingerhoets *et al*., 2013; but see also Helon & Króliczak, 2014) sheds new light on a more general role of the insular cortex. As argued by Paulesu *et al*. (1996), the insula can be considered a ‘bridge’ between Broca’s area (BA44/45) and the supramarginal gyrus (BA40), and its role could be to integrate information from these regions or to ‘converse between the codes’ (p. 152). Similarly to a relay between higher-order and purely motor aspects for language, a comparable role might be performed by this structure for gesture. The insula may, for example, ‘translate’ gesture representations or their visuospatial descriptions encoded in BA40, accompanied by higher-order semantic information encoded in BA44/45, into manual actions executed by the motor cortex. Support for this notion comes from resting-state studies showing rich insular cortex connectivity with the frontal operculum and supramarginal gyrus (Cauda *et al*., 2011). Such a profile of functional connectivity suggests that the insula is an important intermediate structure in a movement execution network. As shown by Eickhoff *et al*. (2009), the insular cortex mediates between Broca’s area and supplementary motor area in language production. It is our contention that the insula plays a similar role for the supramarginal gyrus and premotor cortex. In accordance with this view, potential motor programmes activated in the inferior parietal cortex and selected by the anterior divisions of Broca’s area would need further elaboration with contextual cues before their execution. The insular cortex, potentially bridging many processing centres of the brain, would be a good candidate for this task.

### Directions for further research

Although we observed significant correlations between insular volume or surface asymmetries and functional lateralization of gesture and language, correlations never imply causation. Thus, further studies are required to determine whether an increase in insular volume/surface can be a cause of increased functional lateralization. Furthermore, there is always a possibility that both insular structure and function laterality depends on a hidden cause. Alternatively, the interactions between the two hemispheres before or during gesture and language production might be so complex that the efficiency of the whole process, even though controlled by the insula, would also depend on factors such as the size of the corpus callosum (Josse *et al*., 2008), and the importance of insula may then be overlooked.

From the clinical perspective, our results pointing exclusively to the insula will probably require a provision of further putative structural indices of functional laterality. After all, unless accidently tested before, in a patient during the acute phase of stroke the exact pattern of functional deficits might be hard to assess (cf. Stamenova *et al*., 2010), and the means by which to alleviate them speculative at best (Oliveira & Brito, 2014). Several crucial structural landmarks might therefore be required to help most efficiently choose the proper type, and even the extent, of neurocognitive rehabilitation (cf. Kertesz & Ferro, 1984).

## Conclusions

These outcomes change our understanding of the role of insular structural asymmetries by linking them to the functional lateralization of both gesture and language. Moreover, our findings call for additional investigations, particularly in the gesture domain. Neglected for a long time, the insular cortex is a paramount structure that integrates low-level signals with higher-order motor skills and cognition. Intriguingly, the lateralization of different cognitive functions in the brain may depend on inborn insular asymmetries. Indeed, it is tempting to say that inter-hemispheric differences in the structure of the insular cortex can be used as markers of atypical lateralization of many fundamentally human skills, including gesture and language.

## Abbreviations

aIPS: anterior IPS
ANG: angular gyrus
BA: Brodmann area
cITG: caudal inferior temporal gyrus
CSF: cerebrospinal fluid
FDR: false discovery rate
FusiG: fusiform gyrus
IPL: inferior parietal lobule
IPS: intraparietal sulcus
LI: laterality index
PMd: dorsal premotor cortex
PMv: ventral premotor cortex
rMFG: rostral middle frontal gyri
ROI: region of interest
SMA complex: supplementary and pre-supplementary motor cortices
SMG: supramarginal gyrus
SPG: superior parietal gyrus
